# Influence of inflammatory and metabolic factors on keratoconus risk: a causal inference analysis

**DOI:** 10.1038/s41433-026-04281-y

**Published:** 2026-02-19

**Authors:** Pirro G. Hysi, Alison J. Hardcastle, Alice E. Davidson, Loretta Szczotka-Flynn, Anthony P. Khawaja, Petra Liskova, Sudha Iyengar, Chris J. Hammond, Stephen J. Tuft

**Affiliations:** 1 Department of Twin Research and Genetic Epidemiology, King’s College London, London, UK; 2 Academic Section of Ophthalmology, King’s College London, London, UK; 3 Sørlandet Sykehus Arendal, Arenda, Norway; 4 Department of Ophthalmology, University of Pittsburgh, Pittsburgh, PA USA; 5 UCL Institute of Ophthalmology, University College London, London, UK; 6 Department of Ophthalmology, Case Western Reserve University, Cleveland, OH USA; 7 Moorfields Eye Hospital, NHS Foundation Trust, London, UK; 8 Department of Paediatrics and Inherited Metabolic Disorders, First Faculty of Medicine, Charles University and General University Hospital in Prague, Prague, Czech Republic; 9 Department of Ophthalmology, First Faculty of Medicine, Charles University and General University Hospital in Prague, Prague, Czech Republic

**Keywords:** Risk factors, Corneal diseases

## Abstract

**Background:**

Keratoconus is a complex disease of the cornea, in part caused by environmental exposures whose nature is not fully understood.

**Methods:**

This study relied on previously published results from genome-wide association studies of European ancestry. Summary statistics of available genome-wide association studies on atopy, chronic inflammatory disease and serum glucose levels; results were paired with genomic association results for keratoconus into a well-powered and hypothesis-driven Mendelian Randomisation to statistically test the causal role that may have over keratoconus risk.

**Results:**

Our results confirm a higher risk of keratoconus in patients having any atopic disease (OR = 1.84, 95%CI = 1.52–2.22), but also other chronic inflammatory conditions such as rheumatoid arthritis (OR_IVW_ = 1.11, 95%CI = 1.04–1.18) and Crohn’s Disease (OR_IVW_ = 1.08, 95%CI = 1.02–1.15). Also, higher glucose levels considerably reduced keratoconus risk (OR_IVW_ = 0.49, 95% CI = 0.33–0.72)

**Conclusions:**

Our results suggest that, in addition to atopy, exposure to specific types of systemic inflammatory responses that develop into chronic conditions may increase keratoconus risk. These results will help clinicians to better evaluate the overall keratoconus risk, disease severity and potentially its progression. In conjunction with individual genetic risk profiles, the presence of exposure to atopic disease, high eosinophil counts and comorbidities could have an important predictive role.

## Introduction

Keratoconus is an ectatic disease of the cornea characterised by progressive thinning and distortion that can lead to extreme irregular astigmatism and vision impairment [1]. A recent systematic review of published studies estimated that the global prevalence of keratoconus is 138/100,000 individuals, with wide geographical variation, in part associated with ethnicity [2]. The aetiology of keratoconus is poorly understood, but disease concordance in twins [3] and the occurrence of keratoconus in families [2] suggest that keratoconus has a complex polygenic and environmental origin[4]. A large multi-ethnic genome-wide association study (GWAS) identified several replicable loci that confer susceptibility to keratoconus, which explain a substantial proportion of keratoconus variance in the population [5]. Several exposures not fully under genetic control, such as eye rubbing, atopic disease, and hyperglycaemia, have been suggested as modulators of keratoconus risk [2, 6].

Unlike the genetic effects, the magnitude of risk associated with each non-genetic risk factor, and the presence of causality in their relationship with keratoconus, is uncertain and self-reporting or ascertainment bias can potentially mar observational studies. In addition, there is a strong correlation between the different potential environmental risk factors associated with keratoconus and it can be difficult to differentiate mechanistic disease cascades from non-causal clustering of co-morbidities and behaviours. For example, it is thought that atopic disease is a major risk factor associated with keratoconus [7], as is eye rubbing in the absence of atopy. In this situation, it is unclear whether atopy or eye rubbing, or both, are the cause or of keratoconus, or atopic inflammation of the ocular surface, or visual blur from corneal distortion at all distances, causing discomfort from which patients seek relief through eye rubbing.

Here, we use several large-scale and independent genetic datasets to perform two-sample Mendelian Randomisation (MR), a genetics-powered statistical inference analysis often likened to natural Randomised Clinical Trial experiments [8], to objectively estimate how exposure to different conditions could alter susceptibility to keratoconus. We investigate the causal relationships between the genetic variants associated with the reported exposures (e.g., atopy) and potentially modifiable risks on the development of keratoconus. Importantly, for this analysis, MR does not identify potentially novel disease pathways but objectively confirms suspected mechanisms to overcome some of the limitations of confounding error inherent in observational studies.

## Materials and Methods

The study adhered to the tenets of the Declaration of Helsinki. No specific ethical approval was needed since the analytical models (two-sample MR) were built using instrumental variables selected from previously published summary statistics, including a GWAS of keratoconus [5]. To build the MR models, we incorporated data from six sources, only using the information from populations of European ancestry to avoid confounding arising from trans-ethnic differences in linkage-disequilibrium patterns. We used these data as exposures in several MR models with keratoconus as the MR outcome. We modelled the influence of these exposures on keratoconus by comparing the effect estimates from the exposures with effects of the same SNPs (instrumental variables) in the European samples of keratoconus patients and healthy controls [5].

We selected instrumental variables and estimated effects over serum glucosaemia, as the exposure in the MR models, based on the European participants from the MAGIC Consortium study [9] and the UK Biobank [10]. Models on the risk conferred by atopic diseases and childhood asthma on keratoconus used instrumental variables and effect size estimates from data published by Ferreira et al. [11]. Instruments and effect estimates for asthma were derived from Demenais et al. [12], and analyses for non-asthma-related allergies (atopic dermatitis or allergic rhinitis versus healthy non-atopic controls) from data from Johansson et al [13]. We also built a model that considered the circulating eosinophil count as a marker of the severity of atopic disease [14, 15] and asthma [16, 17]. Data from eosinophilia, as well as other white blood cell counts, were obtained from a previous GWAS [18]. Analyses for rheumatoid arthritis as the exposure was based on the European-ancestry strata of the GWAS published by Ishigaki et al [19] and rheumatoid arthritis [20]. Instruments for inflammatory bowel diseases and their components were identified from the previously published GWAS on these traits [21]. We assumed that common assumptions commonly made on logistic and binary regressions were met in the GWAS results used for the MR analyses, and we did not conduct any post hoc checks to verify the quality of the association analyses.

Only SNPs whose proportion of variance explained for the exposure was higher than that for the outcome were used as instruments [22]. To control for possible weak instruments bias, we used per-SNP and overall F-statistics approximations. Per SNP F-statistics was calculated based on the approximate coefficient of determination (R^2^) and sample as F = R^2^*(N − 1)/(1−R2) as recommended elsewhere [23], where R^2^ = 2*MAF(1-MAF)beta^2, where MAF is the minor allele frequency at each SNP locus used as instrument and beta is the ln(OR) or standardised linear regression coefficient from the summary statistics for the exposure. Additionally, an overall F-statistic was estimated for each MR analysis as described elsewhere [24]. Horizontal pleiotropy was tested by means of an MR-Egger intercept test.

A list of all instruments and the F-statistics is provided in Supplementary Table 1.

We performed the analyses using the ‘*MendelianRandomization*’ package version 0.10.0 in the R statistical environment (www.cran.r-project.org). We evaluated three complementary MR models to assess the causal influence of exposures on the outcome and then selected the inverse-variance weighted method as it provided a balanced and best-powered estimate of the aggregated effect of all instruments on the outcome. These tests are all two-sided. We also provide the results from the simple median and the MR-Egger tests to assess possible undue influence of outliers or unidirectional pleiotropy, respectively.

## Results

We built several primary MR models with keratoconus as the outcome, testing the influence of exposure to fasting glucose levels and atopy, including asthma and other non-asthma atopic and inflammatory diseases. We selected as instruments SNPs that are significantly associated with the exposures as instruments. This included asthma, atopy, eosinophilia, rheumatoid arthritis, inflammatory bowel disease, and glucosaemia. We then compared the effect sizes on these exposures with effect sizes observed in the all-European component of a keratoconus GWAS.

**Fig. 1:**
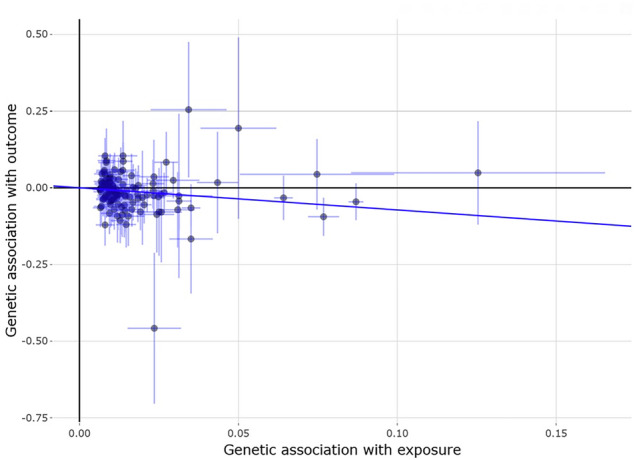
Effect of serum glucose levels on keratoconus. The instruments and the estimates of their effects on the serum glucose levels were obtained from a previously published UK Biobank study [10]. Each point represents the effect sizes of a SNP (instrument) on the exposure (glucose, x-axis) and keratoconus (the “outcome”, y-axis). The non-horizontal trend line illustrates the significant protective effect of glucose levels on keratoconus.

We first compared the information collected on genome-wide significantly associated SNPs influencing fasting time-adjusted glucosaemia exposure from the UK Biobank [10] and the MAGIC consortium [9] (Table 1), with the effects observed in the keratoconus GWAS for the same SNPs (referred to as the instrumental variable in the context of MR studies). In the UK Biobank participants each 1 mmol/L increase of the serum glucose levels causally and significantly reduced the keratoconus risk (OR_IVW_ = 0.49, 95% CI = 0.33–0.72, p = 3.32 × 10^−^^04^, Fig. 1). A similar but slightly stronger protective effect was observed from the analyses of exposures obtained from the MAGIC consortium (OR_IVW_ = 0.43, 95% CI = 0.29−0.64, *p* = 2.32 × 10^−05^), in line with previous observational findings [6].

**Table 1:** Effect of glucosaemia on keratoconus.

	UK Biobank			MAGIC Consortium Study		
Method	Ln(OR)	SE	P-value	Ln(OR)	SE	P-value
Simple median	−0.8639	0.3095	5.25E-03	−0.6747	0.2527	7.58E-03
IVW	−0.7220	0.2013	3.34E-04	−0.8434	0.1993	2.32E-05
MR-Egger	−0.7010	0.3015	0.020	−0.9426	0.3716	0.011
(intercept)	−0.0006	0.0059	0.925	0.0030	0.0095	0.751

Instrument variables and the estimates of their effect size on glycemia were obtained from previously published studies (all glycemia measurements from the UK Biobank glucosaemia study [10] and the fasting glucose levels by the MAGIC Consortium^9^). Ln(OR) refers to changes in keratoconus for each unit increase in glycemia, SE is the standard error of the estimate, P-value are the probabilities estimated from the different models.

**Fig. 2:**
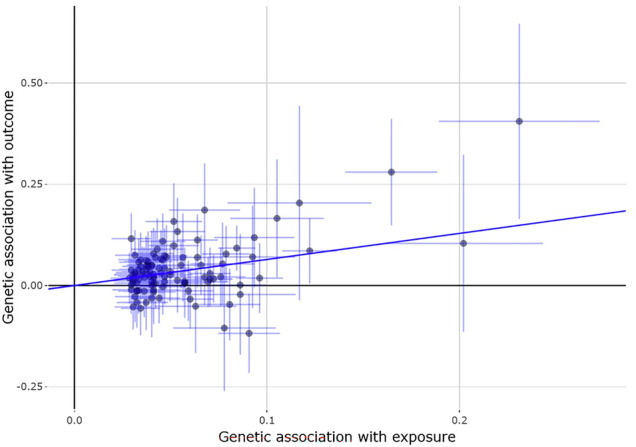
Effect of all atopic diseases combined on keratoconus. Instrument variables and their effect size over atopy were obtained from a previously published GWAS [11]. Each point represents the effect sizes of a SNP (instrument) on the exposure (glucose, x-axis) and keratoconus (the “outcome”, y-axis). The non-horizontal trend line illustrates the significant protective effect of glucose levels on keratoconus.

Conversely, a reported history of any atopic disease, as defined in the genome-wide association studies [11] whose results were used for the selection of instruments, strongly and significantly increased the keratoconus risk (OR_IVW_ = 1.84, 95%CI = 1.52–2.22, *p* = 1.85 × 10^−10^, Fig. 2). This causal association appeared significant, even in the absence of asthma symptoms or diagnosis. A history of atopic disease alone, even when cases of asthma were removed from the analyses, remained a strong causal influence over keratoconus (OR_IVW_ = 1.68, 95% CI = 1.44–1.95, *p* = 1.41 × 10^−11^, Table 2). Due to the absence of adequate published instruments, we could not study the influence of asthma in the absence of other atopic diseases. Using results from published studies, we observed no significant causation for either childhood asthma alone (OR_IVW_ = 1.04, 95% CI = 0.95–1.14), or adult-onset asthma (OR_IVW_ = 1.01, 95% CI = 0.81–1.26) on keratoconus, possibly due to a lack of power.

**Table 2:** Effect of atopy on keratoconus.

Exposure	Method	Ln(OR)	SE	*P*-value
Eosinophil count [18]	Simple median	0.455	0.117	1.03E−04
Eosinophil count	IVW	0.353	0.084	2.49E−05
Eosinophil count	MR-Egger	0.218	0.164	1.85E−01
Eosinophil count	(intercept)	0.005	0.005	3.40E-01
Adult-onset asthma [11]	Simple median	0.013	0.125	0.919
Adult-onset asthma	IVW	0.014	0.112	0.897
Adult-onset asthma	MR-Egger	−0.100	0.375	0.790
Adult-onset asthma	(intercept)	0.009	0.030	0.750
All allergic/atopic diseases combined [11]	Simple median	0.633	0.122	2.12E−07
All allergic/atopic diseases combined	IVW	0.610	0.096	1.85E−10
All allergic/atopic diseases combined	MR-Egger	0.401	0.261	0.12
All allergic/atopic diseases combined	(intercept)	0.011	0.013	0.39
Childhood asthma [11]	Simple median	0.050	0.055	0.362
Childhood asthma	IVW	0.042	0.045	0.353
Childhood asthma	MR-Egger	−0.053	0.107	0.621
Childhood asthma	(intercept)	0.013	0.013	0.331
Non-asthma allergic disease [11]	Simple median	0.476	0.093	3.04E−07
Non-asthma allergic disease	IVW	0.518	0.077	1.41E−11
Non-asthma allergic disease	MR-Egger	0.584	0.192	0.0024
Non-asthma allergic disease	(intercept)	−0.004	0.011	0.71
Asthma [12]	Simple median	0.312	0.118	0.008
Asthma	IVW	0.263	0.091	0.004
Asthma	MR-Egger	−0.169	0.303	0.577
Asthma	(intercept)	0.053	0.035	0.137

The instruments and their effect size estimates were derived from the publications cited in the table. Ln(OR) refers to changes in keratoconus, SE the standard error of the estimate, *P*-value are the probabilities estimated from the different models.

Different definitions of atopic disease used in each of the original GWAS from which the instruments were selected.

To minimise self-reporting bias, we built a model considering circulating eosinophil counts [18] as exposures, as they are an objectively measured parameter often considered a marker of severity of the severity of atopic disease [14, 15] or asthma [16, 17]. Consistent with previous results for atopy, elevated circulating eosinophil counts were a causal risk for keratoconus (OR_IVW_ = 1.42, 95% CI = 1.21–1.68, *p* = 9.27 × 10^−^^06^, for each eosinophil counted per microliter, Table 2). No significant effects were observed for other white blood cells studied in that GWAS (data not shown).

Finally, to test the hypothesis that the presence of other common chronic inflammatory conditions alters keratoconus risk, we modelled the risk of keratoconus as a function of exposure to rheumatoid arthritis and inflammatory bowel disease (Ulcerative colitis and Crohn’s disease). The MR analyses found a weaker, although statistically significant, causal association with rheumatoid arthritis (OR_IVW_ = 1.11, 95%CI = 1.04−1.18, *p* = 0.003). Interestingly, given the early onset of keratoconus, juvenile arthritis did not show any statistically significant causation on keratoconus (Supplementary Table 2). We observed a weak, but statistically significant causal effect of Crohn’s Disease on keratoconus (OR_IVW_ = 1.08, 95%CI = 1.02–1.15, *p* = 0.005) but not ulcerative colitis (OR_IVW_ = 1.015, 95%CI = 0.95–1.09). The inflammatory bowel disease overall did not causally associate with keratoconus (OR_IVW_ = 1.05, 95%CI 0.98–1.11, Supplementary Table 2), possibly due to the lack of effect of ulcerative colitis. No significant outlier effects or pleiotropy were detected in any of the analyses reported in this study, and there was no suggestion of weak instrument bias in any of our results (Supplementary Table 1).

## Discussion

Although there is interest in genetic factors that predispose to keratoconus, the contribution of environmental risk factors to this disease has been difficult to quantify. In this study, we used large genetic datasets from different sources to perform two-sample MR to objectively assess the potential non-genetic influences on the development of keratoconus. Our study results support the view that atopic diseases or traits considerably increase keratoconus risk. Keratoconus risk also increases proportionally with increasing degrees of genetically determined peripheral eosinophilia, a biomarker of atopic disease. Our results also suggest that rheumatoid arthritis and some forms of inflammatory bowel disease, potentially in their earliest stages, are positively and causally associated with keratoconus. A lack of statistically significant evidence for juvenile arthritis causality on keratoconus should be interpreted with caution due to the small sample size and less precise effect estimates for that exposure. Our results raise the possibility that susceptibility to systemic immune-mediated disorders and not just localised behavioural reactions to atopy in conjunctival tissues, such as eye rubbing, may increase the keratoconus risk.

Eye rubbing is often reported to be more common in patients with keratoconus than in unaffected controls [25–27]. Mechanical stress from eye rubbing was thought to cause immediate thinning of the corneal epithelium and distortion of the cornea [28] as well as keratocyte loss, and the rubbing action may release proteinases, among others, in the corneal stroma that trigger corneal thinning [29]. However, eye rubbing is a common symptom in ophthalmic conditions such as dry eye, visual deprivation, or allergic rhinoconjunctivitis, and the stimulus for rubbing may be independent of atopy [7]. While it would have been interesting to directly study the influence of mechanical friction and eye rubbing, such tests could not be conducted due to a lack of genome-wide association studies for these traits and the absence of suitable instruments.

Asymptomatic immunological findings can precede clinically manifest rheumatoid arthritis by a decade [30, 31] and often result from imbalances in Th1/Th2 effector choices [32]. Observational studies show that rheumatoid arthritis, atopy [33, 34] and inflammatory bowel disease [35] are weakly correlated. The hypothesis of a direct immune action in keratoconus merits further attention. In addition to bronchial asthma and other atopic diseases, keratoconus shares part of its genetic risk with inflammatory bowel disease [5], which may support the hypothesis that immune changes contribute to keratoconus pathogenesis. The levels of several pro-inflammatory molecules, including IL-6 and TNF-α, are elevated in the tears [36, 37] and excised corneal tissue [38] of patients with keratoconus.

The protective effect of higher fasting levels of the glucosaemia in the general population supports previous observations of a negative correlation between diabetes and keratoconus [39] and changes in corneal biomechanics as a result of hyperglycaemia are reported [40]. This effect is likely mediated by non-specific and non-enzymatic cross-linking reaction between proteins and sugars in the corneal stroma that stiffens the cornea. This phenomenon (Maillard reaction) results in lysine and arginine glycation when there is persistent hyperglycemia [41, 42]. Twin studies show that both atopy [43, 44] and hyperglycaemia [45, 46] are determined in part by both genetic and environmental factors. However, the protective effects of high glucosaemia on keratoconus do not outweigh the damage caused elsewhere in the body.

We also present evidence that higher levels of glucose reduce the risk of keratoconus. Although a similar model and results have been reported before, the previous work [47] the calculated effects used multi-ethnic panels for keratoconus, which often limits the reliability and precision of the estimates.

The causal inference analyses reported here allow us to estimate the risk conferred by mechanisms independent of the heritable risk factors that contribute to keratoconus susceptibility. Current genetic models based on the high-frequency genetic risk factors associated with keratoconus correctly predict a large proportion of keratoconus heritability [5]. Additional knowledge on non-genetic factors, such as atopy and higher levels of fasting glucosaemia, would refine existing predictive models and improve the accuracy of individual predictions. Our results need to be interpreted in the context of the advantages and limitations inherent in the study design. MR are unbiased statistical models which establish the causality of environmental factors over a disease using independent genetic instruments, which, as per Mendel’s second law on independent assortment, are inherited randomly, much like the random assignment of participants in the arms of randomised clinical trials. They are, however, limited in their ability to characterise mechanisms of action and they rely on the critical assumption that there is a direct causal pathway between exposure and outcome. Like any other statistical test, MR results need to be interpreted with caution because of the possibility of bias and unconfounding. In this manuscript, we have attempted to follow best practice (reviewed elsewhere [48]) in the hope of averting their most common sources. Other and less obvious sources of bias, beyond what is discussed here, may affect results [49]. Most importantly, theoretically, any factor associated independently with both exposure and keratoconus could act as a confounder. MR analyses also rely on a number of assumptions that are critical to achieving truthful results. Although the variable instruments selected for this study are clearly appropriate (robust genetic evidence for genetic associations with exposures), independence and exclusion restriction assumptions cannot be tested statistically. Therefore, these results depict true causality as long as these assumptions are met. The instruments selected appeared strong (F-statistics » 10), which suggests that the potential for weak instrument bias is low. Caution is, however, required when interpreting the causal effects for these exposures, since they may vary depending on the ethnic group mind in which they are estimated, the case-control ratio, or, in certain case,s due to the “winner’s curse” phenomenon, which could affect MR effect sizes.

Finally, we analysed the potential effects of multiple exposures, which raises the possibility of multiple testing issues. A full Bonferroni-type correction is, however, impossible, due to both the non-independence of some of these exposures (asthma, atopy, and chronic inflammatory conditions) and the different power for each of these analyses due to differences in exposures and GWAS sample sizes. Because we conducted 12 such tests, any IVW results with *p* < 0.0042 (0.05/12) remain significant after Bonferroni correction, although the true significance threshold is almost certainly less conservative than this value.

In conclusion, these results provide the first objective estimates of keratoconus risk arising from higher glucosaemia, atopic disease, and related blood biomarkers. We anticipate this will be the first in a series that characterises the effects of various exposures that are not directly genetic nor entirely environmentally driven in the pathogenesis of keratoconus. As more data becomes available, future studies will provide more genetic instrument variables that will empower higher-resolution causal inference analyses and functional biomolecular experiments will be needed to independently verify these effects and study their underlying causes. Supplemental material is available at Eye’s website.

## Summary

### What was known before

- This article explores the effect that two sets of common exposures, glycemia and chronic inflammatory disease have on keratoconus risk.
- High glycemia confers protection from keratoconus developments
- Atopic disease and possibly other chronic inflammatory conditions increase susceptibility to keratoconus.

### What this study adds

- This study establishes a direct causal connection between atopic disease and keratoconus. These results suggest that inflammatory processes rather than rubbing in response to eye irritation and itching are responsible for the changes in the cornea.

## Supplementary information

Supplementary Table 1

Supplementary Table 2

## Data Availability

All work described in this article was conducted on freely and publicly accessible information, available from the GWAS Catalogue (https://www.ebi.ac.uk/gwas/) and the original publications cited in the References.
